# The Length and Number of Sedentary Bouts Predict Fibrinogen Levels in Postmenopausal Women

**DOI:** 10.3390/ijerph17093051

**Published:** 2020-04-28

**Authors:** Pascal Izzicupo, Andrea Di Blasio, Andrea Di Credico, Giulia Gaggi, Anastasios Vamvakis, Giorgio Napolitano, Fabrizio Ricci, Sabina Gallina, Barbara Ghinassi, Angela Di Baldassarre

**Affiliations:** 1 Department of Medicine and Aging Sciences, University “G. D’Annunzio” of Chieti-Pescara, 66100 Chieti and Pescara, Italy; 2 3rd Department of Internal Medicine, Papageorgiou Hospital, Aristotle University of Thessaloniki, 54124 Thessaloniki, Greece; 3 Department of Neuroscience, Imaging and Clinical Sciences, University “G. D’Annunzio” of Chieti-Pescara, 66100 Chieti and Pescara, Italy; 4 Department of Clinical Sciences, Lund University, 21428 Malmö, Sweden

**Keywords:** hemostasis, cardiovascular disease risk, sedentary behavior, physical activity, women’s health, menopause

## Abstract

Menopause is associated with adverse changes in coagulation homeostasis. We aimed to investigate the association between objectively measured sedentary behavior (SB) and SB bouts (i.e., number and length of SB bouts) vs. fibrinogen levels in post-menopausal women. Fifty-three post-menopausal women (age 59.8 ± 6.2 years, BMI 27.3 ± 4.4) wore a multisensory device (Sensewear Mini Armband, BodyMedia, Inc., Pittsburgh, PA) for 5 days, to measure SB and physical activity (PA). Blood samples were collected to measure serum fibrinogen. Fibrinogen was directly correlated with SB (r = −0.48, *p* < 0.01), lying down during awake time (r = −0.50, *p* < 0.01), and both medium (11–30 mins) and very long bouts (>1 h) of SB (r = −0.59, *p* < 0.01; r = −0.51, *p* < 0.01, respectively), and inversely correlated with moderate to vigorous-intensity physical activity (r = −0.39, *p* < 0.01). Furthermore, fibrinogen was also directly correlated with BMI (r = −0.28, *p* < 0.05). In postmenopausal women without prevalent cardiovascular disease, the number of prolonged and uninterrupted sedentary bouts is directly correlated with increased fibrinogen levels, regardless of PA and BMI. This result suggests the importance of delivering new strategies to counteract the increase of sedentariness and inactivity of the postmenopausal population.

## 1. Introduction

In recent years, sedentary behavior (SB) has emerged as a new, independent behavioral risk factor for many noncommunicable diseases (NCDs) [1,2]. SB reflects the time spent while sitting, reclining, or lying down during waking hours, yielding overall energy expenditure ≤1.5 METs [3]. SB differs from physical inactivity, which represents the failure to achieve recommended levels of physical activity (PA) for a given population [4,5]. Accordingly, an individual can be physically active, but still spending a large amount of daily time in SB. Although PA is a powerful protective tool against the onset and progression of most NCDs, there is evidence that SB determines harmful effects through mechanisms acting independently of PA levels [6]. Pooled data from more than one million individuals showed higher mortality in active people with a high amount of SB than in non-active people with a lower amount SB. Nevertheless, very large amounts of PA have been shown to compensate for excessive sedentary time [7]. Furthermore, experimental evidence suggests that frequent breaks from SB is associated with similar or even better effects than standard moderate-to-vigorous intensity training session (e.g., cycling 1 hour) on a large spectrum of cardiovascular risk factors and physiological parameters [8].

Cardiovascular disease has a low incidence in premenopausal women. However, loss of ovarian hormones during perimenopause and menopause leads to a sharp increase in CVD [9]. Indeed, dysregulation of circulating pro-inflammatory cytokines and coagulation homeostasis commonly occurs during the menopausal transition, and play a key role in the initiation and progression of CVD [10,11]. In particular, plasma fibrinogen levels are noticeably higher in women, especially after menopause [12]. Considering the well-known effect of prolonged uninterrupted/cramped sitting on the activation of the coagulation system [13], recent studies showed an independent association between SB and several inflammatory and hemostatic biomarkers, regardless of PA levels and body composition [14,15,16,17,18,19].

However, little is known on the relationship between SB and hemostatic parameters in postmenopausal women. Few studies correlated SB with inflammatory/hemostatic markers, including fibrinogen in middle-aged and older individuals, with mixed results concerning the role of sex [14,16,17,18]. Briefly, middle-aged women showed higher levels of plasma fibrinogen than men [17], and, after adjustment for sex, the correlation between fibrinogen and SB remained significant only for women in two cohorts [14,17]. Although participants were of menopausal age, these studies did not mention menopausal status, but, presumably, the effect of sex might be due at least partially to it [12]. Furthermore, in these researches, neither PA nor SB were objectively assessed. Only two studies explored the association between inflammatory/hemostatic markers and objectively assessed SB, in older men [20] and patients with intermittent claudication [21]. We sought to explore the correlation between objectively measured SB and circulating fibrinogen levels in postmenopausal women, a population particularly susceptible to increase the risk of CVD due to changes in hemostatic parameters [10,11,12,13]. Our hypothesis is that fibrinogen correlates with daily time spent in SB and with the number and duration of SB bouts, regardless of PA and health-related anthropometric measures.

## 2. Materials and Methods 

### 2.1. Participants

The description of the participant characteristics and the screening and testing procedures for this study have been described in detail previously [22,23]. Briefly, female participants volunteered from the local population of the municipalities of Chieti and Pescara (Italy) between January and May 2009. A telephone interview was used to determine whether these women met the inclusion criteria, of: Age <65 years old; no estrogen-replacement therapy; no endocrine disease; no history of diabetes mellitus or hypertension, dyslipidemia, and cardiovascular disease; no dieting or use of nutritional supplements; no antihypertensive, hypolipidemic and antithrombotic drugs; no participation in any regular exercise program during the 2 years prior to the study. Post-menopause was defined as the time after which a woman has experienced at least 12 consecutive months of amenorrhea without a period, also accompanied by plasma estradiol < 20 pg/ml. Overall, 68 women met the criteria to be included in the study. Of these, 53 had valid physical activity and sedentary behavior assessment, as described below (sufficient number of days and minutes per day). The Ethics Committee of the ‘G. D’Annunzio’ University of Chieti–Pescara approved this study, and all of the participants gave their written informed consent. The study was conducted in accordance with the Declaration of Helsinki.

### 2.2. Study Design

Measurements were performed at controlled conditions in terms of temperature (21–23 °C) and humidity (50%). Participants presented after overnight fasting, without having performed maximal muscle exertion the day before. They underwent medical history data collection, physical examination, blood sampling, and anthropometry. Subsequently, they received the SenseWear mini armbands (BodyMedia, Pittsburgh, USA), a device able to assess objective daily physical activity and SB profiles and were instructed to wear it for 5 consecutive days in a free-living context, except for water-based activities (e.g., shower, swimming).

### 2.3. Blood Sampling and Analysis

After 8 h overnight fasting, 15 ml venous blood samples were collected in evacuated tubes that were pre-treated with a silica gel clot activator (Vacuette, Greiner Bio-One). The whole blood was allowed to clot for 20 min at room temperature, and the clot was then removed by centrifugation at 2000× *g* for 10 min at room temperature. The serum was separated from the clot and stored at −80 °C prior to analysis. Plasma fibrinogen and estradiol were determined using enzyme-linked immunosorbent assays (ELISAs; DRG International Inc., Mountainside, NJ, USA), according to the manufacturer’s suggestions. All of the samples were processed in duplicate during the same assay session. The intra-assay coefficient of variation was <5%, and the inter-assay precision was <8%. Optical densities were read on a standard plate reader at 450 nm (SpectraMax 190; Molecular Devices, Sunnyvale, USA).

### 2.4. Anthropometry

A first-level Anthropometrist of the International Society for the Advancement of Kinanthropometry carried out the body measurements of the participants in their fasting condition. Body mass and stretched stature were measured to the nearest 0.1 kg and 0.1 cm, respectively, with the participants dressed in light clothing and without shoes, using a stadiometer with a balanced-beam scale (Seca 220; Seca, Hamburg, Germany). The participant BMIs were calculated as body mass/stature^2^ (kg/m^2^). Anthropometric tape (Cescorf, Porto Alegre, Brasil) was used to measure waist circumference, as the smallest circumference between the rib cage and the iliac crest at the end of normal expiration.

### 2.5. Daily Physical Activity and Sedentary Behavior Profiling

Daily PA was measured under free-living conditions for 5 consecutive days, which included 3 weekdays and at least 1 weekend day, using SenseWear mini armbands [24,25,26]. The participants wore their monitors all through the 5 measurement days, except while bathing. No raining days were present among the recorded periods. To consider the recordings valid, the wear time criteria were at least 600 min/day with at least 3 valid weekdays and a valid weekend day. It has been shown that 3 weekdays of recording using the SenseWear Pro3 Armband were enough to achieve a reliability of 0.80 for SB, LIPA, MIPA, PAL, and energy expenditure, in middle-aged men and women [27]. The SenseWear mini armband is based on the same technology, but a 3-axis accelerometer is used instead of the 2-axis version in the SenseWear Pro3 [28]. The internal algorithms are slightly different between the monitors. However, SenseWear mini armband showed slightly better performance over the SenseWear Pro3 [28]. The SenseWear mini armband integrated the information gathered by the 3-axis accelerometers and sensors (i.e., skin and near-body temperature, heat flux, galvanic skin response) with the sex, age, stature, weight, smoking status, and handedness of the user, using the SenseWear Professional 8.0 software (BodyMedia, Pittsburgh, USA). It has been shown that combining accelerometry with physiological parameters can improve the accuracy of measurement [24,26,28]. The SenseWear mini armband provided extensive information about wear time, daily PA (e.g., intensity, number of daily steps, energy expenditure), and other behaviors such as sleep, SB, and lying down. In particular, SB was recognized as the activities with an intensity lower than 1.5 METs [25], whereas lying down was identified considering the orientation of the horizontal axis according to the manufacturer’s algorithm. The SenseWear mini armband was worn on the left arm, over the triceps. This, had been shown able to furnish reliable recording with respect to other placements (i.e., wrist, hip, ankle), and in respect to both other devices and placements [26], in both health and pathological conditions [24,25], according to the manufacturer’s validation.

From the recorded data, the focus in the present study was on time spent on PA according to 3 intensity levels: An intensity >3 METs and ≤6 METs (i.e., moderate-intensity physical activity; MIPA); an intensity >6 METs and ≤9 METs (i.e., vigorous-intensity physical activity; VIPA); and an intensity >9 METs (i.e., very vigorous-intensity physical activity; VVIPA). The total time spent above 3 METs was considered as MVPA. Since it was well recognized that the efficacy of MIPA may be higher when accumulated in bouts of at least 10 minutes [29,30], we focused on the number of PA bouts of at least 10 minutes episodes (nPA_10_), as well on the cumulative and average length of these bouts (cPA_10_, aPA_10_, respectively). In addition, SB, the time spent in light-intensity physical activities (LIPA), and the time spent lying down while awake (from here on lying time) were considered. Since SenseWear mini armband cannot distinguish between sitting and standing, SB was considered as the whole time spent in physical activities with an intensity ≤1.5 METs, excluding nocturnal sleeping, while lying time was considered as the SB spent lying down, as a subset of total SB. The LIPA was the time spent in physical activities >1.5 METs and ≤3 METs. The number of daily bouts of SB that lasted less than 5 consecutive minutes (SB_veryshort_), between 6 and 10 minutes (SB_short_), 11 and 15 minutes (SB_11–15_), 16 and 20 minutes (SB_16–20_), 21 and 30 minutes (SB_21–30_), 31 and 45 minutes (SB_31–45_), 46 and 60 minutes (SB_46–60_), 1 and 2 hours (SB_1–2_ h), and more than one (SB_verylong_) were calculated using a specifically written application. The application first recognized consecutive bouts of SB, then grouped them according to the desired duration and finally counted the number of bouts for each desired duration. Furthermore, we collapsed SB bouts between 11 and 30 minutes (SB_medium_), and between 31 and 60 minutes (SB_long_).

### 2.6. Statistical Analysis

Data were initially tested for normality with the Shapiro–Wilk statistic and presented as mean ± SD. Pearson’s correlation coefficient was applied to explore the correlation among health-related anthropometric measures, PA and SB profiles, and fibrinogen. Because MIPA was not normally distributed, log-transformation was applied (logMIPA) before to perform Pearson’s correlation analysis. Sedentary bouts longer than 2 hours were not inserted in the correlation analysis because their occurrence was not frequent (Table 1). Partial correlation, including MVPA and BMI as covariates, was further performed to explore the independent effect of SB on fibrinogen. Furthermore, to explore the independent effect of SB fragmentation on fibrinogen from health-related anthropometric measures, PA and total time spent in sedentary pursuits, a partial correlation analysis including BMI, MVPA, and SB as covariates were also performed. Finally, multiple linear regression with backward elimination was performed to define the best predictors of fibrinogen levels among anthropometric measures, PA, SB, and SB fragmentation. The variables included in the regression equation were chosen based on the correlation coefficients and from different categories: BMI for weight category, MVPA for PA (because it also included VIPA and the correlation coefficient was higher than logMIPA and PAB10c), lying time for SB, and both SB_medium_ and SB_verylong_ for SB fragmentation. SPSS 12.0 (SPSS^®^, Chicago, IL, USA) was used for data analysis. The level of significance was set at *p* ≤ 0.05. 

## 3. Results

### 3.1. Basal Characteristics, Physical Activity, and Sedentary Behavior Profiles

The physiological and anthropometrical characteristics of the participants and their PA and SB profiles are presented in Table 1. Participants were overweight and at increased risk for cardiovascular and metabolic diseases, based on BMI, WC, and WC to stature ratio. Participants were on average active with a mean daily MIPA and cPA_10_ exceeding 100 minutes and 30 per day, respectively. The involvement in VIPA was almost null, with a mean daily value of 1 minute. Most of the awake time was spent in sedentary pursuits and LIPA (Table 1). On the other hand, sleep time (mean 6 hours per night), was at the lower limit of the recommended levels for adults [31]. Participants spent, on average, two bouts of SB per day between 30 minutes and 1 hour and at least one bout per day longer than 1 hour (Table 1).

### 3.2. Fibrinogen, Physical Activity, and Sedentary Behavior Profiles

Fibrinogen was directly correlated with weight, BMI, SB, lying time, SB_medium_, and SB_verylong_ (Table 2). Specifically, fibrinogen correlated with SB_11–15_, SB_16–20_, SB_21–30_ minutes, and SB_1–2_ h. The correlation was weak for fibrinogen vs. weight, BMI, and SB_16–20_ and moderate for fibrinogen vs. SB, lying time, SB_medium_, SB_verylong_, SB_11–15_, SB_21–30_ minutes, and SB_1–2_ h. Furthermore, a moderate, inverse correlation between fibrinogen and MVPA, cPA_10_, and logMIPA was found, as well as a tendency for an inverse correlation with LIPA. No correlation was found between WC, WC to stature ratio, and sleep with fibrinogen (Table 2). Furthermore, no correlation was found between SB_short_ (including SB_≤5_ and SB_6–10_) and SB_long_ (including SB_31–45_ and SB_46–60_) vs. fibrinogen. Since a direct association existed between SB and health-related anthropometric measures, while both MIPA and VIPA were inversely associated with several cardiometabolic risk factors, including BMI, the correlation analysis between SB and lying time vs. fibrinogen was also performed including BMI and MVPA as covariates. A moderate correlation was confirmed also after controlling for BMI and MVPA (r (49) = 0.334, *p* = 0.017; r (49) = 0.379, *p* = 0.006, respectively). Furthermore, after controlling for BMI and MVPA, a moderate correlation was confirmed also for SB_medium_ and SB_verylong_ (r (49) = 0.492, *p* = 0.001; r (49) = 0.369, *p* = 0.008, respectively). In particular, a weak and moderate correlation was also confirmed, respectively, for SB_11–15_ (r (49) = 0.297, *p* = 0.034) and both SB_21–30_ (r (49) = 0.385, *p* = 0.005) and SB_1–2_ h (r (49) = 0.311, *p* = 0.026). However, after controlling for BMI, MVPA, and SB, a moderate correlation was confirmed only for SB_medium_ (r (48) = 0.416, *p* = 0.003). Specifically, a weak correlation was also confirmed for SB_11–15_ (r (48) = 0.280, *p* = 0.049), and SB_21–30_ (r (48) = 0.273, *p* = 0.05). 

### 3.3. Predictive Equation for Fibrinogen Based on Anthropometric Measures, Physical Activity, and Sedentary Behavior Profiles

A significant model (F (2, 50) = 19.93, *p* < 0.001) predicted 42.1% of the sample outcome variance (Adj. R^2^ = 0.421). Two predictors were entered into the model: SB_medium_ (β = 8.24, t = 4.01, *p* ≤ 0.001) and SB_very-long_ (β = 15.14, t = 2.92, *p* = 0.05) were significantly associated with higher fibrinogen levels. Lying time, SB, and BMI were excluded from the model.

## 4. Discussion

In this study we reported a direct correlation between fibrinogen levels and the length and number of sedentary bouts, in particular with SB_medium_ and SB_very-long_ (uninterrupted sedentary bouts between 11 and 30 min and above 1 hour, respectively), in a cohort of postmenopausal women, regardless of BMI and MVPA. Medium SB bouts showed a moderate significant correlation with fibrinogen even after adjustment for total daily SB. In our cohort, the the length and number of sedentary bouts was the strongest predictor of fibrinogen levels, regardless of weight category and total time spent in both PA and SB. 

CV risk sharply increases after menopause and changes in hemostatic parameters have an important role [32]. Indeed, plasma fibrinogen levels are higher in women [17] than in men and further increase after menopause [12]. In this regard, both biological—e.g., lack of estrogens—and behavioral factors—e.g., decrease in PA levels [33]—are established risk factors for CVD after menopause. However, previous evidence regarding the relationship between SB and fibrinogen in women are scarce and only based on non-objective assessment of PA and SB. Furthermore, previous studies were focused on the general population, without any distinction concerning menopausal status. 

Two prospective cohort studies examined the longitudinal association between TV viewing (a key indicator of SB) and several inflammatory/hemostatic markers, including fibrinogen, during a 23-year and 4-year follow-up in British men and women, respectively, 44 [16] and over 50 years old [18]. Both studies found a correlation between TV viewing time and fibrinogen, even after controlling for sex and other covariates. Conversely, another two cross-sectional studies focused on male and female middle-aged participants found a correlation between fibrinogen and TV viewing time in women but not in men. In 55 ± 12 years old participants this association was independent of leisure-time PA, waist circumference, and high-sensitivity C-reactive protein, and there was no interaction with abdominal and overall adiposity [14]. On the other hand, in 44–45 years old participants, the association was attenuated after adjustment for BMI [17]. Our study is in line with studies including participants of similar age and older, in which the association is independent of health-related anthropometric measures and PA components. However, the lack of consistency among studies might be due to difference in study design, PA assessment, and population. In particular, we focused only on postmenopausal women while previous studies involved middle-aged women. Although they were on average of menopausal age, the proportion of pre- and post-menopausal women in these cohorts is unknown, and this may have played a role on the different effects of sex among studies. Furthermore, the aforementioned studies used various subjective methods to assess SB and its indicators, while in the present study SB was assessed objectively. It is worth noting that most of the studies which found an association with inflammatory/hemostatic parameters used TV viewing as an indicator of SB, an approach that has been recently questioned [34]. Indeed, studies in adults indicated that TV viewing was poorly correlated to sitting time [35,36,37], while it reflects other factors such as socioeconomic status and mental health that are determinants of poor health [38,39]. This aspect is also evident in relation to hemostatic/inflammatory parameters, since most correlate with TV viewing but not to sitting at work [17].

Only two previous studies explored the association between hemostatic markers and SB through objective assessment, in older men [20] and patients with symptoms of peripheral arterial disease [21]. In the study of Farah et al., SB remained associated with fibrinogen after adjustment for sex, age, PA status, BMI, and severity of peripheral arterial disease [21]. Parsons et al. found an association between sedentary time and some fibrinolytic markers but they did not assess fibrinogen [20]. Furthermore, they did not find any association with sedentary bouts. In our study, we found a direct moderate correlation between fibrinogen and both SB_medium_ and SB_very-long_ that was independent of MVPA and BMI. After adjustment for total time of SB, the correlation was maintained for SB_medium_, whereas the predictive model included both SB_medium_ and SB_very-long_ while it excluded BMI, MVPA, and lying time (a subset of SB with stronger correlation than SB with fibrinogen in this study). This result suggests that the SB pattern is a determinant for fibrinogen levels in postmenopausal women, more than total SB time. In other words, the interruption of prolonged SB can mitigate its negative effects. Our findings are in line with previous results from an experimental trial, which found that increases in plasma fibrinogen after uninterrupted prolonged sitting were attenuated by active interruptions [15]. However, our study is the first to our knowledge that examined this aspect with objective assessment in a free living context.

An interesting finding of the present study is that fibrinogen correlates with lying time but not with sleep. These two behaviors are characterized by the same postural (lying or reclining) and intensity (< 1.5 METs) elements but differ for the consciousness status. Various metabolic risk factors are affected by the induction of 5 days of bed rest, an extreme model of SB, including microvascular dysfunction, although does not affect inflammatory markers [40]. Considering also that the association between TV viewing and hsCRP is affected by WC while remains for fibrinogen, and that in the present and previous studies [14,17,18] the association between fibrinogen and SB and its components was independent of or still significant after adjusting for BMI or WC, it is possible that sitting and lying time induce microvascular dysfunction through an impairment in the hemostasis that is independent of inflammation and adiposity, which explains the association between SB and fibrinogen. Indeed, it has been experimentally demonstrated that bending and compressing the lower limbs strongly affect vascular function [6], although it remains to be elucidated why this seems to not apply to sleep. Furthermore, it has been previously demonstrated that the amount of muscle mass involved during exercise can affect circulating inflammatory and vascular factors [41,42] and the same principle might apply to body surface involved during SB that is inactive and compressed. Indeed, in the present study, the association between lying time and fibrinogen was slightly higher than with non-lying down SB. Considering also the difficulty in distinguishing between standing and sitting when using wrist- and waist-worn accelerometers, this result suggests exploring this eventuality through rigorous experimental designs, which can furnish convincing biological evidence on the mechanism by which SB harms health irrespective of those that induce the beneficial effect of PA, such as cardiovascular remodeling [43], antioxidant protection [44,45], and improved endothelial function [46,47]. The latter, together with hemostatic changes shown in this study and others [15], seems to be a coherent mechanism but there is also evidence that does not support the independent effects of SB, since sitting-induced endothelial dysfunction is prevented by an exercise bout [6].

The relationship between fibrinogen and SB observed in the present study reinforces the idea of reducing and interrupting SB, especially in postmenopausal women who show higher plasma fibrinogen [12,17] and tend to spontaneously reduce PA levels [33]. However, a series of considerations must be done in order to translate these results into practical applications. To date, the evidence for both an independent unhealthy effect of too much sitting and the benefits of breaking SB are still inconclusive, in spite of a number of national guidelines, which recommend reducing and interrupting as much as possible SB [48,49,50]. In particular, the lack of consistency between sitting and TV time in epidemiological studies and the impossibility of distinguishing between standing and sitting if not using thigh-worn sensors make questionable the adoption of quantitative recommendations on SB. The results of the present study, together with the results of Howard et al. (2013) [15], seems to suggest that limiting prolonged SB and including light physical activities every 30 minutes or even less should attenuate the relationship between SB and fibrinogen, as well as other markers of cardiovascular and metabolic risk. Furthermore, the present study enriches the result of the aforementioned study by providing evidence in a free-living context, which easier translates in long term outcomes compared to the acute and responses of short-term experimental designs. However, as recently suggested by Stamatakis et al. (2019) [34], the adoption of the term stationary behavior instead of SB should be more pertinent, considering that waist- and wrist-worn accelerometers do not completely distinguish between standing and sitting and the same principle applies for SenseWear mini armband. Thus, part of the sedentary time might just be the lack of ambulatory activity instead of sitting. This consideration implies that quantitative indications on SB based on the results of the present and previous studies are still premature. This idea should be further explored in studies with a longitudinal design, possibly positioning sensors on different body sites.

### Strength and Limitations

A major strength of the present study is the objective assessment of PA and SB, which allows to study their fragmentation [29,30,51], avoiding the overestimation of PA and the underestimation of SB that commonly applies to subjective methods [52]. Furthermore, while previous studies explored the relationship between fibrinogen and SB in a laboratory setting and in pathological population, our study investigated this association in a free-living setting and for the first time in post-menopausal women. Previous evidence indicates that women are more prone to show high fibrinogen than men [12,20]. Furthermore, after menopause, women decrease PA levels [33]. Nonetheless, the indication of frequent sitting interruption is reasonable for both genders.

There are some limitations to our study. Firstly, domains of SB were not explored in our research. It has been shown that SB domains are important in the determination of the levels of inflammatory/hemostatic markers. Indeed, while adverse effects are associated with TV viewing, it doesn’t seem to apply to sitting at work. Investigating SB and PA domains in study with objective measurement may provide further insights, especially in researches with a large variance of PA levels, like in the present study. Secondly, although SenseWear mini armbands (BodyMedia, Pittsburgh, USA) provides accurate measurement of different activity intensity [24,26,28] its body positioning is not ideal to discriminate between standing and sitting, like positioning on the thigh [34]. This means that SB in our study might be overestimated. Finally, our study lacks important measures of cardiovascular function and morphology, such as carotid intima-media thickness [53], ankle-brachial index [54] and vascular function [55]. However, all the participants underwent a medical examination, which included resting ECG and a maximal exercise stress test [22,23].

## 5. Conclusions

In postmenopausal women, SB_medium_ and SB_very-long_ are associated with procoagulant profile that could predispose to a higher risk of vascular disease, while frequent interruptions prevent these harmful effects. Although our sample was on average very active, the correlation between fibrinogen and SB remained moderate even after considering PA levels. This result suggests the importance of delivering new strategies to counteract the increase of sedentariness and inactivity of the population. The use of wearable devices seems promising [56] while the adoption of quantitative protocols aimed to interrupt SB seems still premature.

## Figures and Tables

**Table 1:** Characteristics of the sample.

N = 53	Mean ± SD
Age (years)	59.8 ± 6.2
Weight (kg)	65.5 ± 11.1
BMI	27.3 ± 4.4
Waist circumference (cm)	86.2 ± 11.8
Waist to stature ratio (cm)	0.6 ± 0.1
Resting heart rate	66.1 ± 12.2
Resting systolic blood pressure	127.5 ± 12.8
Resting diastolic blood pressure	79.8 ± 6.4
Blood glucose	84.18 ± 13.7
Total cholesterol	225.1 ± 42.0
HDL	64.6 ± 15.2
LDL	138.8 ± 36.9
Triglyceride	106.4 ± 57.7
SB (min)	591.1 ± 132.3
lying time (min)	130.9 ± 112.9
LIPA time (min)	356.1 ± 114.5
MIPA time (min)	101.1 ± 82.5
VIPA time (min)	1.1 ± 2.9
MVPA time (min)	102.1 ± 83.5
Sleep time (min)	367.2 ± 69.2
SB_veryshort_ (num)	37.4 ± 11.6
SB_short_ (num)	7.4 ± 2.6
Sedentary Bouts 11–15 min (num)	3.7 ± 1.7
Sedentary Bouts 16–20 min (num)	2.4 ± 1.1
Sedentary Bouts 21–30 min (num)	2.5 ± 1.3
SB_medium_ (num)	8.6 ± 2.7
Sedentary Bouts 31–45 min (num)	1.9 ± 1.2
Sedentary Bouts 46–60 min (num)	1.1 ± 0.8
SB_long_ (num)	2.9 ± 1.6
Sedentary Bouts 1–2 h (num)	1.1 ± 0.9
SB_verylong_ (num)	1.2 ± 1.0
Sedentary Bouts > 2 h (num)	0.2 ± 0.3

BMI: Body mass index; MIPA: Moderate-intensity physical activity; LIPA: Light-intensity physical activity; VIPA: Vigorous-intensity physical activity; MVPA: Moderate to vigorous-intensity physical activity; SB_veryshort_: Sedentary bouts ≤ 5 minutes; SB_short_: Sedentary bouts between 6 and 10 minutes; SB_medium_: Sedentary bouts between 11 and 30 minutes; SB_long_: Sedentary bouts between 31 and 60 minutes; SB_verylong_: Sedentary bouts longer than 1 hour; Sedentary Bouts > 2 h: Sedentary bouts longer than 2 hours.

**Table 2:** This table shows the correlation between anthropometric, physical activity, and sedentary behavior variables with fibrinogen levels.

Variables	Correlation with Fibrinogen (Pearson’s r)
Weight (kg)	0.28 *
BMI	0.30 *
WC (cm)	0.20
Waist to stature ratio (cm)	0.20
SB (min)	0.48 **
lying time (min)	0.50 **
LIPA (min)	−0.24
logMIPA (min)	–0.39 **
VIPA (min)	–0.12
MVPA (min)	–0.39 **
Sleep (min)	0.11
SB_veryshort_ (num)	0.20
SB_short_ (num)	0.04
Sedentary Bouts 11–15 min (num)	0.37 *
Sedentary Bouts 16–20 min (num)	0.30 *
Sedentary Bouts 21–30 min (num)	0.50 **
SB_medium_ (num)	0.59 **
Sedentary Bouts 31–45 min (num)	0.20
Sedentary Bouts 46–1 h (num)	0.08
SB_long_ (num)	0.19
Sedentary Bouts 1–2 h (num)	0.45 **
SB_verylong_ (num)	0.51 **
nPA_10_ (num)	–0.27
cPA_10_ (min)	–0.32 *
aPA_10_ (min)	–0.14

BMI: Body mass index; WC: Waist circumference; SB: Sedentary behavior; LIPA: Light-intensity physical activity; MIPA: Moderate-intensity physical activity; VIPA: Vigorous-intensity physical activity; MVPA: Moderate to vigorous-intensity physical activity; SB_veryshort_: Sedentary bouts ≤ 5 minutes; SB_short_: Sedentary bouts between 6 and 10 minutes; SB_medium_: Sedentary bouts between 11 and 30 minutes; SB_long_: Sedentary bouts between 31 and 60 minutes; SB_verylong_: Sedentary bouts longer than 1 hour; nPA_10_: The number of PA bouts of at least 10 minutes; cPA_10_: Cumulative length of bouts of at least 10 minutes; aPA_10_: Average length of bouts of at least 10 minutes. df (51); * *p* < 0.05; ** *p* < 0.01.
